# CD133 Expression in Normal Skin and in Epithelial Cutaneous Tumors

**DOI:** 10.1155/2013/385604

**Published:** 2013-09-12

**Authors:** S. H. Nam-Cha, R. Serrano-Vargas, E. Escario, J. M. Azaña, R. Calero-Oliver, A. G. Martín, E. Poblet

**Affiliations:** ^1^Department of Pathology, University General Hospital, 03003 Albacete, Spain; ^2^AECC Translational Oncologic Research Unit, University General Hospital, 03003 Albacete, Spain; ^3^Department of Dermatology, University General Hospital, 03003 Albacete, Spain; ^4^Regulation of Cell Growth Laboratory, Inbiomed Foundation, 20009 San Sebastian, Spain; ^5^Department of Pathology, University General Hospital and Murcia University, 30003 Murcia, Spain

## Abstract

*Background*. Expression of human CD133 (human prominin-1) in cancer cells has been postulated to be a marker of stemness and is considered as a putative marker of cancer stem cells (CSCs). We designed a study to describe the expression pattern of CD133 in normal skin and in epithelial cutaneous neoplasms. *Methods*. The CD133 immunohistochemical expression of forty-three eccrine and apocrine tumors was compared to that observed in other epithelial tumors of the skin. In addition, flow cytometry was used to detect the CD133 expression of four epithelial skin neoplasms, including one porocarcinoma. *Results*. CD133 immunoreactivity at the apical or at the apicolateral surface of cells forming glandular structures was observed. Cells from solid areas of benign or malignant tumors were not stained. The porocarcinoma derived culture cells showed a 22% of CD133 positive cells using flow cytometry, while squamous cell carcinoma cultures contained less than 0.1%. *Conclusions*. These observations indicate that CD133 is a specific marker of glandular differentiation that could be included in the diagnostic panel of cutaneous tumors with possible eccrine or apocrine differentiation. However, the use of CD133 expression as a marker of CSCs should be interpreted with caution in experiments of skin.

## 1. Introduction

In the last few years, a growing body of evidence has been reported supporting the notion that the capability to sustain tumor formation and growth exclusively resides in a small population of cells called cancer stem cells (CSCs). Search of markers that demonstrate the stem cell-like phenotype of these tumor initiating cells is an active field of investigation, and several stem cell markers have been described recently in skin tumors [1, 2].

CD133 is the human homologue of mouse prominin-1, a five transmembrane domain cell surface glycoprotein that has received notable attention because of its expression restricted to various somatic stem cell subpopulations [3]. This surface antigen was discovered as a target of a monoclonal antibody defined as Mab AC133, a marker expressed by a subpopulation of CD34 positive hematopoietic stem cells derived from human foetal liver and bone marrow [3–5]. Subsequently CD133 was detected in different human normal tissues, including kidney, prostate, brain, and pancreas, and found to be specifically associated with microvilli and other plasma membrane protrusions [6–9]. Moreover, this antigen has also been identified in haematological malignancies and in several solid human neoplasms, including glial tumors, prostate carcinoma, kidney carcinoma, hepatocarcinoma, colorectal carcinoma, and malignant melanoma [7, 9–13]. The aim of many of these studies was to determine the existence of CSCs, defined as a small group of cancer cells that have the capacity for self-renewal, tumor initiation, and tumor growth maintenance. Antibodies routinely used for purification of AC133-positive cells target poorly characterized glycosylated epitopes of uncertain specificity. Although the functional activity of CD133 is still controversial and its physiological function is not yet determined [14], it is becoming clear that this cell surface protein is a unique marker of both plasma membrane protrusions and membrane microdomains [3]. It has been suggested that in this cellular location CD133 may act as a regulator of membrane lipid composition or it can participate in the mechanisms of cell polarity and migration [15].

In previous studies that report the CD133 immunohistochemical staining of human glandular epithelia, such as salivary glands, lacrimal glands, pancreatic ducts, endocervical glands, and sweat glands, a peculiar pattern of CD133 expression has been described [8, 9, 14]. In these epithelia, CD133 has been shown to be localized to plasma membrane protrusions at the apical areas of polarized cells. A similar CD133 apical cytoplasmic staining pattern of cells surrounding a lumen has also been observed in carcinomas from ovary, colon, or pancreas [14, 15].

The finding of the CD133 antibody staining sweat gland cells prompted us to design a study that we now report, in order to evaluate the expression of CD133 in skin eccrine and apocrine tumors.

## 2. Materials and Methods

### 2.1. Cases

A retrospective search retrieved 43 previously diagnosed skin eccrine and apocrine adnexal tumors from the files of the Department of Pathology of the Albacete University Hospital. Glass slides, paraffin blocks, and histopathological reports from all the cases were obtained. Slides were reviewed and adnexal skin tumors were diagnosed according to the criteria of the World Health Organization (WHO) classification [16], including eccrine spiradenoma (*n* = 3), nodular hidradenoma (*n* = 5), eccrine hidrocystoma (*n* = 3), poroma (*n* = 6), porocarcinoma (*n* = 1), syringocystadenoma papilliferum (*n* = 3), chondroid syringoma (*n* = 4), syringoma (*n* = 6), cylindroma (*n* = 4), hidradenoma papilliferum (*n* = 2), apocrine hidrocystoma (*n* = 3), microcystic adnexal carcinoma (*n* = 2), and apocrine carcinoma (*n* = 1). Cases of basal cell carcinoma (*n* = 5) and squamous cell carcinoma (*n* = 4) were also included in this study to compare the results obtained in these neoplasms with those obtained in sweat gland tumors. To evaluate the CD133 expression in normal skin, we analyzed skin from different areas obtained from the margins of six surgically resected tumors of the skin and from three autopsies. 

 For culture of skin cancer cells, seven samples from human skin tumors that corresponded to one eccrine porocarcinoma and six squamous cell carcinomas were obtained. Fresh tissue from these cases was included in Dulbecco's Modified Eagle's medium (DMEM), supplemented with penicillin/streptomycin and fungizone, immediately after surgical resection.

### 2.2. Immunohistochemistry

Formalin fixed, paraffin embedded tissue sections 4 *μ*m wide were cut, deparaffinized in xylene, and rehydrated in a graded series of ethanol. Endogenous peroxidase activity was blocked with 3% H_2_O_2_ for 5 minutes. Slides were treated with heat-induced epitope retrieval and immunostained with three different anti-CD133 monoclonal antibodies: AC133 monoclonal antibody, 293C3 monoclonal antibody, and AC141 monoclonal antibody (all from Miltenyi Biotec, Germany). Antibodies were tested at different dilutions and times of incubation. CD133 detection was performed by using the EnVision system-HRP (Dako, Glostrup, Denmark) in a DakoCytomation Autostainer platform, according to the manufacturer's instructions. Diaminobenzidine (DAB substrate system, Dako, Glostrup, Denmark) was used as chromogen. From the three different anti-CD133 antibodies tested, only one, the AC133 monoclonal antibody, worked in paraffin. The other two antibodies tested failed because sections incubated with these antibodies showed no staining or because the immunostaining observed when high concentrations of the antibodies were used was considered unspecific (similar staining was observed in negative controls). The AC133 monoclonal antibody gave reliable results on the paraffin embedded sections tested. We establish for this antibody a 1 : 10 dilution and 40 minutes of incubation at room temperature as the preferred working conditions. Subsequently all the experiments were performed in those conditions. 

Sections of skin with normal sweat glands were used as positive controls. In addition, when present in the sections, normal sweat glands from the skin adjacent to the neoplasms were used as internal positive controls. Negative controls were performed by omitting the anti-CD133 antibody during the primary antibody incubation. 

Immunohistochemical staining of solid areas and of acinar or ductal structures from the tumors was separately evaluated. CD133 staining was graded using a semiquantitative scale. Acinar or ductal structures were evaluated as follows: (−) no staining; (+) staining of secretory material in the lumen of isolated ductules or acini and/or weak staining of the apical or luminal border of few ductules or acini; (++) clear staining of the apical or luminal border of most ductules or acini present in the tumour; (+++) staining of the apical or luminal border of all ductules or acini present in the tumor. CD133 expression was evaluated by two senior pathologists (EP and SYNC) in a blinded fashion without knowledge of clinical and pathological information. In cases of discrepant assessments, slides were reevaluated by both pathologists under a multihead microscope, and an agreement was obtained. 

### 2.3. Culture of Cancer Cells from Skin Tumors

Tumor fragments were mechanically and enzymatically disaggregated by digestion with collagenase type IA (2 mg/mL) (Gibco, Invitrogen) in Hank's balanced salt solution (HBSS) at 37°C for 2 hrs. Cells were filtered through a 40 *μ*m nylon mesh and were further dissociated by serial passage through serological pipettes. Digested tissue was centrifuged at 1000 ×g for 10 min and the pellet washed several times to obtain a single cell suspension. Isolated cells were plated into culture flasks and grown at 37°C in DMEM/F12 media containing 10% fetal bovine serum and supplemented with penicillin/streptomycin and fungizone.

### 2.4. Flow Cytometry Analysis

All experiments were performed on cell suspensions prepared at first passage of primary culture from tumours. Cells were detached using 0.02% EDTA in phosphate-buffered saline (PBS) for 15 min at 37°C and washed with PBS before staining. Cell suspensions were adjusted to 1 × 10^6^ cells/mL and incubated with the recommended antibody dilution (1/10) for 20 min in the dark (4°C). Samples without primary antibody were used as negative controls. The antibody used was anti-CD133/1 (AC133)-APC (Miltenyi Biotec). Unbound antibodies were removed by washing with PBS, and cells were resuspended in a suitable volume of buffer. Analysis was performed on a LSRII flow cytometer (BD Biosciences). Data were analyzed using Summit V5.0.1. 5170 software (Dako). 

## 3. Results

### 3.1. Clinical Findings

 The patient cohort consisted of 29 males and 23 females, ranging in age from 24 to 90 years (median, 49 years). The site of presentation was variable, most of them localized on the head and neck region. Clinical data are summarised in Table 1. All patients underwent excisional skin biopsy.

### 3.2. Immunohistochemical Findings

From the three different monoclonal antibodies tested on formalin-fixed, paraffin- embedded tissue sections, and in accordance with previous reports [7], only the AC133 gave sensitive and reliable results. The overall immunohistochemical staining pattern observed with this antibody was closely related to tissue architecture. The staining was mainly localized to the apical surface of cells that formed ductal or glandular structures. Immunohistochemical findings observed in these structures using the anti-CD133 antibody are summarized in Table 1 and are described below. Solid areas from any of the adnexal tumors examined or from the basal cell and squamous cell carcinomas tested failed to demonstrate CD133 positivity. 

#### 3.2.1. Normal Tissue

**Figure 1:**
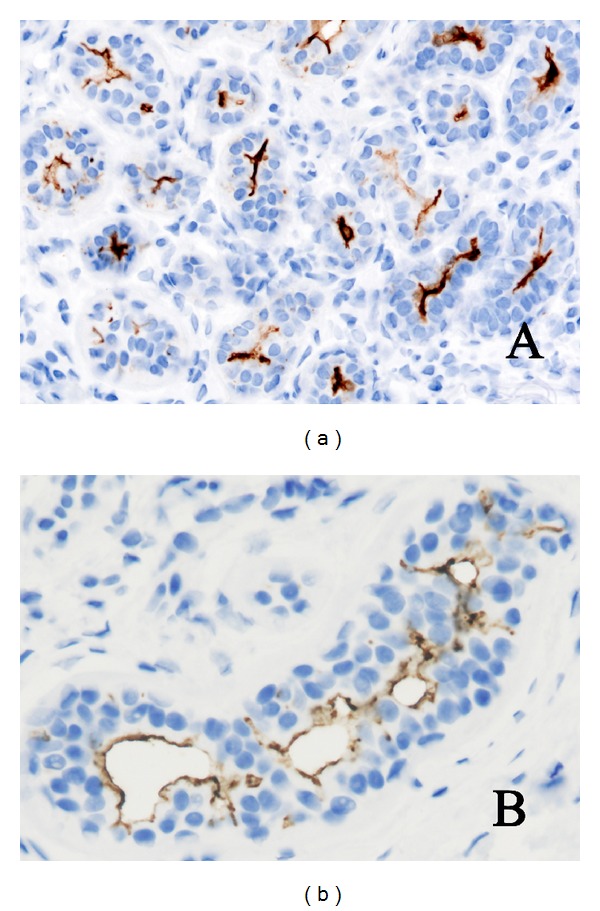
CD133 in normal eccrine glands stains the endoluminal surface of the cells and the sweat gland secretion (a). Intercellular canaliculi at the lateral membrane of eccrine cells are also observed (b).

 CD133 expression was observed at the endoluminal or apical surface of the cells of the acini and terminal ductules of normal eccrine glands (Figure 1(a)). Intercellular canaliculi formed at the lateral membrane of eccrine cells located in the secretory portion of normal sweat glands were clearly stained (Figure 1(b)), as well as the secretion found in the lumen of eccrine ducts. Staining of normal apocrine glands at the terminal ductules was observed at the apical border of the cells, although with a patchy distribution. In acinar areas of these glands, where the apocrine decapitation secretion was obvious, CD133 showed only a weak or negative staining.

#### 3.2.2. Tumors

 CD133 was not expressed in any of the squamous or basal cell carcinomas tested. Using the AC133 antibody the adnexal tumors analyzed in this work showed the following staining pattern (summarized in Table 1).

**Figure 2:**
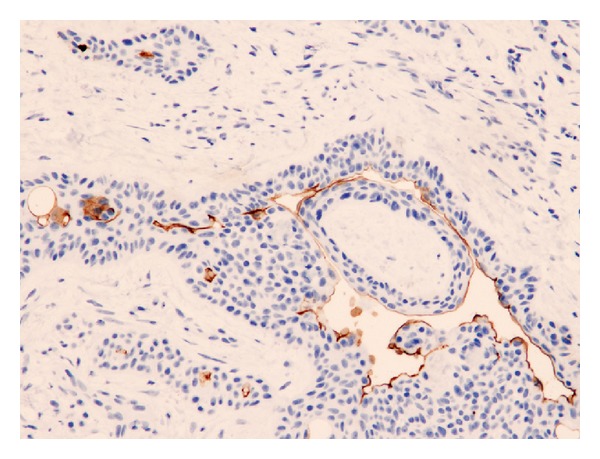
CD133 positivity at the inner surface of chondroid syringoma branching tubules. No staining of the stromal cells or of outer epithelial cells is noted.

**Figure 3:**
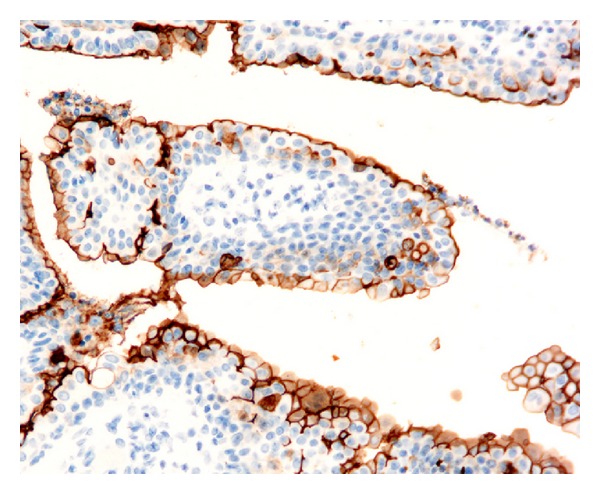
Papillary projections of syringocystadenoma papilliferum lined by pseudostratified columnar epithelium are CD133 positive. The positivity is very intense at the endoluminal surface.

**Figure 4:**
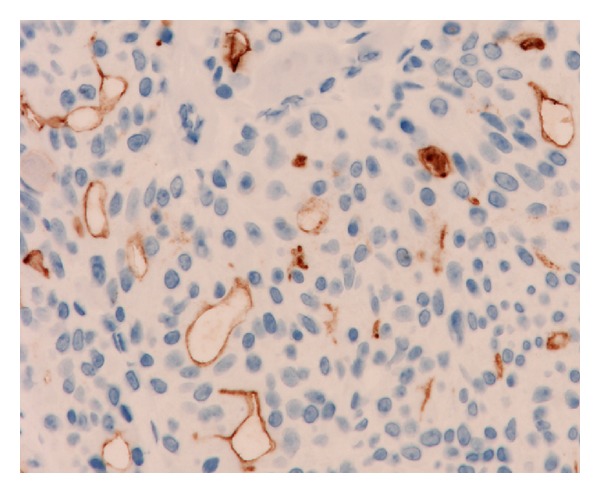
Linear CD133 staining of columnar cells lining tubules of a hidradenoma case. Only cells that are located at the inner surface of the tubules are stained.

*Benign Tumors with Eccrine or Apocrine Differentiation. *All the eccrine spiradenoma cases presented CD133 immunoreactivity at the apical membrane of the duct-like structures present within the tumor nodules. The luminal surface of the epithelial cells forming the unilocular cysts of all the eccrine hidrocystoma cases analyzed was also positive. Ducts and small cysts present in three of the six eccrine poroma cases showed intense staining of the internal cell layer from the proliferated tubules, and this staining was located at the endoluminal border of the cells. The other three poroma cases analyzed showed the same staining pattern in most tubules, but with a (++) or (+) staining pattern of CD133 positivity. Duct-like structures, present in the four cylindroma cases, showed also immunoreactivity at the apical membrane of the epithelial cells. The staining of the four chondroid syringomas was prominent, and all the ductules present, which formed occasional branching structures, showed intense positivity at the apical membrane (Figure 2). The dilated and tortuous ducts, which lead into cystic spaces of all the syringocystoadenoma papilliferum cases, showed immunoreactivity at the apical portion of the epithelial cells or at the luminal surface of the cysts, with an intensity that ranged from (++) to (+++) (Figure 3). The five cases diagnosed as nodular hidradenoma showed isolated duct-like structures which were positive for CD133 with an apical staining of the cells that formed the tubules (Figure 4) that ranged in intensity from (++) to (+++). The two hidradenoma papilliferum cases showed also expression of CD133 at the apical portion of the glandular structures. Only two of the six syringoma cases tested showed consistent staining at the luminal surface of the small ducts that formed the tumors, which were lined usually by two layers of cubical epithelial cells. The apocrine hidrocystoma cases failed to demonstrate any positive immunoreactivity.

**Figure 5:**
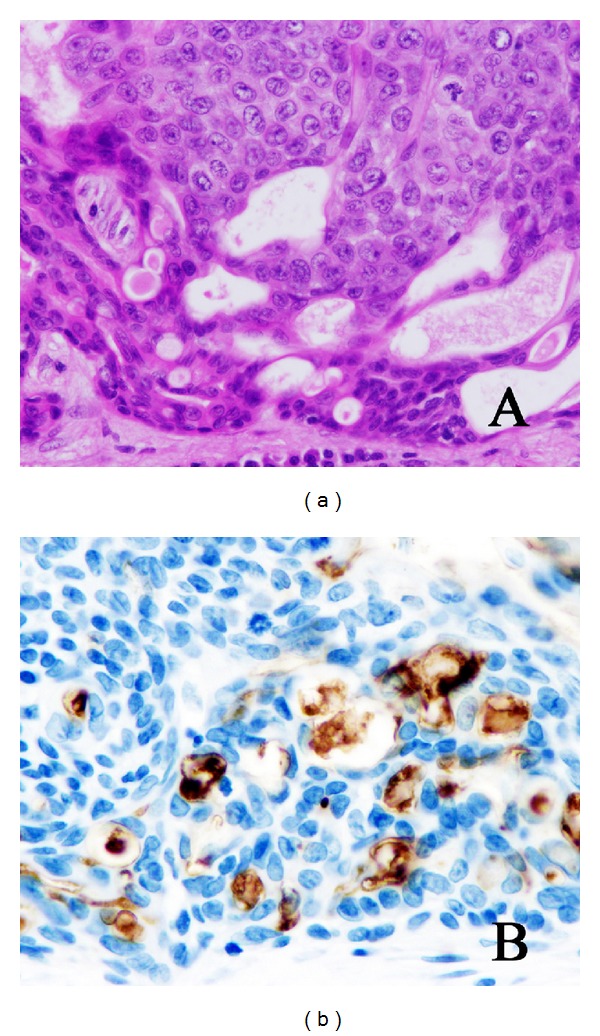
Ductal structures of porocarcinoma seen in H&E sections (a) are CD133 positive (b).

*Malignant Tumors with Eccrine or Apocrine Differentiation. *No immunoreactivity could be demonstrated at the luminal surface of the cysts of the case diagnosed as apocrine carcinoma. The two microcystic adnexal carcinomas analyzed in this study showed strong CD133 positivity at the apical surface of cells that formed the small and large lumina of the tubular structures. CD133 immunostaining could be demonstrated at the very atypical cells that surrounded the tubular structures of the porocarcinoma case (Figure 5). 

### 3.3. Characterization of Primary Cultures from Human Skin Tumors and Flow Cytometry Analysis

 Seven samples derived from human skin tumors were processed with the goal of obtaining single cell suspensions. Due to an insufficient number of cells obtained from the tumors or to fungal or bacterial contamination, only four samples were successfully cultured and analyzed for cell surface CD133/1 epitope expression. These successfully cultured biopsies include one eccrine porocarcinoma and three squamous cell carcinomas. Microscopic examination of the cultured cells revealed different morphologies that varied consistently to the histological tumor type. Cells from squamous cell carcinoma showed epithelioid or spindle cell appearance while eccrine porocarcinoma derived cells had more rounded shape and grouped together into islets of small and atypical cells. The presence of epithelial cells in these cultures was confirmed through E-cadherin and EpCam positive expression as epithelial markers.

**Figure 6:**
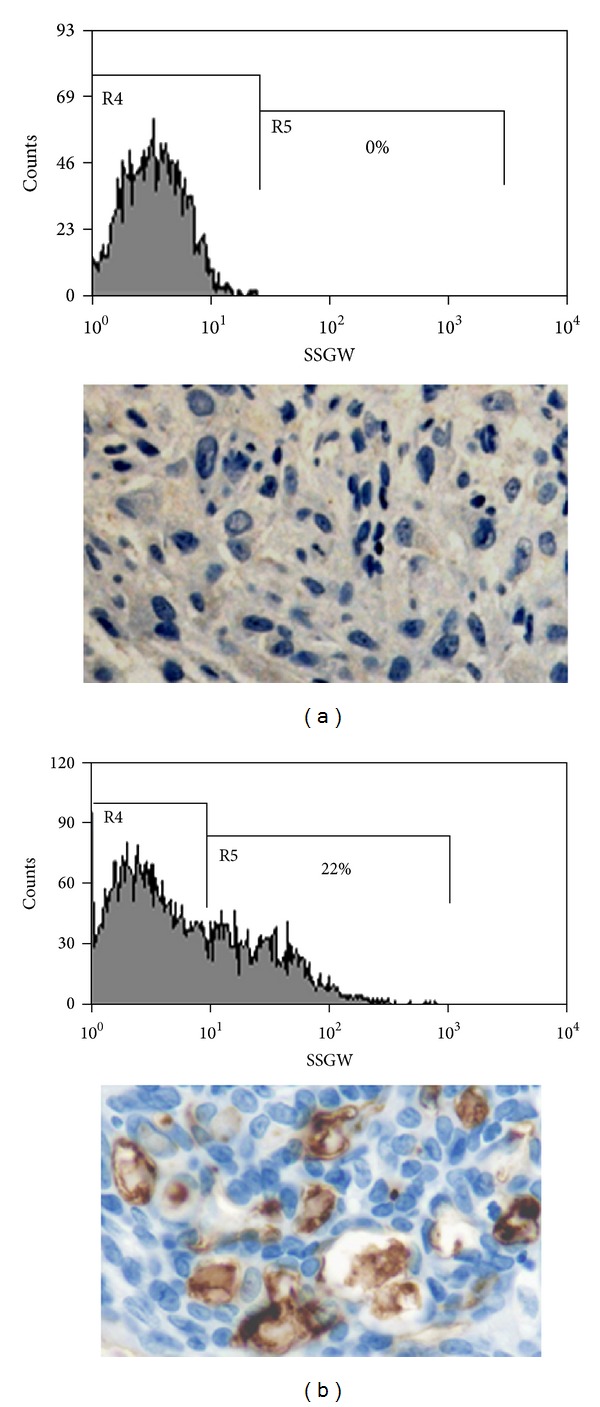
CD133 expression profiles in human skin primary tumour cells. Analysis of CD133/1 expression in samples from eccrine porocarcinoma (b) and from squamous cell carcinoma (a). Flow cytometry of porocarcinoma derived cell cultures show a 22% of CD133/1 epitope positive cells and CD133 immunostaining. Squamous cell carcinoma shows negative staining for CD133 and 0% positive CD133 cells on flow cytometry.

To determine whether skin tumor cell cultures contained a population of CD133 positive cells, we used flow cytometry to examine the expression of CD133/1 surface marker with the AC133 antibody. All samples from squamous cell carcinoma contained less than 0.1% of positive cells, while nearly 22% of positive cells were detected in eccrine porocarcinoma.  Figure 6 shows representative histograms from the two skin tumor types evaluated.

## 4. Discussion

Progress in cutaneous stem cell biology, and in carcinogenesis understanding, depends heavily on the availability of markers specific for distinctive stem cell populations [1]. Known markers of skin stem cells [17, 18] or of stem cells that have been identified in other organs or tumors [19] may be used for detection of cutaneous CSCs. One of these markers that can be tested in the skin is the CD133 protein (prominin-1). The first data reporting CD133 expression using the monoclonal antibody AC133, produced against a novel stem cell glycoprotein antigen, showed that CD133 was selectively expressed on CD34 hematopoietic stem and progenitor cells derived from human fetal liver and bone marrow, as well as from blood [2, 3]. Since then, several reports have shown that prominin-1 can be used to identify and isolate human stem cells from various sources, including the hematopoietic system [3], the prostate [7], the pancreas [9], or the kidney [6]. In addition, monoclonal antibodies against CD133 have been used for the identification and isolation of a putative population of tumor-initiating cells or CSCs in a number of human carcinomas [9, 11, 20] and in malignant melanoma [12]. In glioma, the increased number of CD133 positive cancer cells, as well as the presence of clusters of these cells, has been proposed as a significant prognostic factor, independent of other factors like tumor grade [21]. However, other studies, using different and novel anti-CD133 clones, have suggested that the expression of CD133 is not limited to stem and progenitor cells and seems to be expressed in adult epithelial cells of mouse and human tissues [8, 11, 14, 22, 23]. For instance, using a CD133 clone, called 13A4, derived from mouse prominin-1, Weigmann et al. [8] demonstrated expression of prominin-1 at the apical surface of neuroepithelial cells and adult kidney proximal tubules, and Karbanová et al. [14], using a monoclonal antibody (80B258) generated against a human prominin-1 polypeptide, observed immunoreactivity in adult human tissues, particularly in glandular epithelia. These results indicate the need for further studies to clarify whether the AC133 antigen, previously found in flow cytometry to be restricted to the CD34 positive progenitor cells, is expressed in adult epithelial cells [4, 5, 24, 25]. It has been suggested that the variable CD133 expression observed is related to the differential affinity of the diverse antibodies to various glycosylated forms of CD133 [24]. The monoclonal antibody AC133 recognizes a glycosylated epitope of prominin-1 [4], and, interestingly, the glycosylation of this protein may change depending on the state of cellular differentiation, or it can be altered during the process of malignant transformation [11, 22, 24]. In our study on human skin tumors, we have observed that CD133 expression is greatly dependent on the formation of sweat gland ductules and sweat gland secretion. CD133 immunoreactivity was mainly seen at the apical/endoluminal surface of duct-like structures or cysts of most benign and malignant eccrine tumors. None of the tumors studied, neither benign nor malignant, presented isolated or small clusters of CD133 positive neoplastic cells in solid areas. Similar CD133 staining pattern of the apical cell membrane of secretory cells has been previously reported in normal tubular structures such as kidney proximal tubules [8] or in tumor glands of colorectal cancer [20]. CD133 staining was also found at the apical/endoluminal surface of cells forming a lumen in ovarian carcinomas [26]. In these cases, the apical location of CD133 has been implicated in a possible secretory function of this molecule. The morphological distribution of the CD133 staining demonstrates a correlation with well recognized secretory structures of normal tissues and glands, suggesting a functional relation of CD133 with secretion. In fact, in our study, tumors suspected to be originated in the ductal areas of the sweat glands, like syringomas, showed a less intense pattern of staining than those originated at the acinar portions. 

The CD133 expression on the cell surface of differentiated tumor cells is an argument against CD133 as a specific cancer stem cell marker in the skin. However, the existence of a small CD133^+^ CSC population cannot be completely ruled out, as it has been demonstrated in other organs, where CD133 is expressed on the cell surface of both, CSCs, and differentiated tumor cells [9, 14, 27]. 

Taken together, our findings demonstrate a specific immunohistochemical expression of the AC133 antibody at the secretory surface of differentiated normal and neoplastic cells of eccrine glands. Experiments that use this antibody as a marker of CSCs in skin should be interpreted with caution. The AC133 antibody is a sensitive and specific marker of cutaneous glandular differentiation, useful for the diagnosis of tumors with possible eccrine or apocrine differentiation. 

## Figures and Tables

**Table 1:** Summary of clinical and immunohistochemical findings.

Diagnosis	Age	Location	CD133
Eccrine spiradenoma (*n* = 3)	76	Nasal	+++
	52	Neck	+++
	58	Nasal	++

Hidradenoma (*n* = 5)	38	Unknown	+++
	77	Forehead	+++
	85	Face	+++
	48	Head	++
	78	Unknown	++

Eccrine hidrocystoma (*n* = 3)	76	Nasal	+++
	52	Neck	++
	58	Nasal	+++

Poroma (*n* = 6)	34	Plantar	++
	85	Forearm	+++
	51	Scalp	+++
	31	Leg	+
	59	Unknown	+++
	67	Unknown	++

Porocarcinoma (*n* = 1)	90	Back	+++

Syringocystadenoma papilliferum (*n* = 3)	36	Scalp	++
	60	Pectoral	+++
	28	Scalp	+++

Syringoma (*n* = 6)	42	Unknown	+
	36	Forearm	++
	32	Neck	+
	31	Eyelid	+
	67	Inferior eyelid	−
	36	Eyelid	++

Cylindroma (*n* = 4)	51	Scalp	++
	51	Scalp	+++
	80	Scalp	++
	47	Unknown	+++

Hidradenoma papilliferum (*n* = 2)	75	Vulvar	+
	31	Vulvar	+

Chondroid syringoma (*n* = 4)	38	Supralabial	++
	36	Nasal	+++
	60	Forehead	+++
	53	Eyelid	+++

Apocrine hidrocystoma (*n* = 3)	33	Eyelid	−
	79	Eyelid	−
	40	Face	−

Apocrine carcinoma (*n* = 1)	62	Axilar	−

Microcystic adnexal carcinoma (*n* = 2)	46	Upper lip	+++
	53	Upper lip	+++

CD133 staining was graded using a semiquantitative scale. Acinar or ductal structures were evaluated as follows: (−) no staining; (+) staining of secretory material in the lumen of isolated ductules or acini and/or weak staining of the apical or luminal border of few ductules or acini; (++) clear staining of the apical or luminal border of most ductules or acini present in the tumour; (+++) staining of the apical or luminal border of all ductules or acini present in the tumor.

## References

[1] Sellheyer K (2011). Basal cell carcinoma: cell of origin, cancer stem cell hypothesis and stem cell markers. *British Journal of Dermatology*.

[2] Sellheyer K, Nelson P, Krahl D (2009). Dermatofibrosarcoma protuberans: a tumour of nestin-positive cutaneous mesenchymal stem cells?. *British Journal of Dermatology*.

[3] Bauer N, Fonseca A-V, Florek M (2008). New insights into the cell biology of hematopoietic progenitors by studying prominin-1 (CD133). *Cells Tissues Organs*.

[4] Miraglia S, Godfrey W, Yin AH (1997). A novel five-transmembrane hematopoietic stem cell antigen: isolation, characterization, and molecular cloning. *Blood*.

[5] Yin AH, Miraglia S, Zanjani ED (1997). AC133, a novel marker for human hematopoietic stem and progenitor cells. *Blood*.

[6] Sagrinati C, Netti GS, Mazzinghi B (2006). Isolation and characterization of multipotent progenitor cells from the Bowman’s capsule of adult human kidneys. *Journal of the American Society of Nephrology*.

[7] Richardson GD, Robson CN, Lang SH, Neal DE, Maitland NJ, Collins AT (2004). CD133, a novel marker for human prostatic epithelial stem cells. *Journal of Cell Science*.

[8] Weigmann A, Corbeil D, Hellwig A, Huttner WB (1997). Prominin, a novel microvilli-specific polytopic membrane protein of the apical surface of epithelial cells, is targeted to plasmalemmal protrusions of non-epithelial cells. *Proceedings of the National Academy of Sciences of the United States of America*.

[9] Immervoll H, Hoem D, Sakariassen P, Steffensen OJ, Molven A (2008). Expression of the “stem cell marker” CD133 in pancreas and pancreatic ductal adenocarcinomas. *BMC Cancer*.

[10] Wang S, Xu ZY, Wang LF, Su W (2013). CD133^+^ cancer stem cells in lung cancer. *Frontiers in Bioscience*.

[11] Florek M, Haase M, Marzesco A-M (2005). Prominin-1/CD133, a neural and hematopoietic stem cell marker, is expressed in adult human differentiated cells and certain types of kidney cancer. *Cell and Tissue Research*.

[12] Klein WM, Wu BP, Zhao S, Wu H, Klein-Szanto AJP, Tahan SR (2007). Increased expression of stem cell markers in malignant melanoma. *Modern Pathology*.

[13] Suetsugu A, Nagaki M, Aoki H, Motohashi T, Kunisada T, Moriwaki H (2006). Characterization of CD133^+^ hepatocellular carcinoma cells as cancer stem/progenitor cells. *Biochemical and Biophysical Research Communications*.

[14] Karbanová J, Missol-Kolka E, Fonseca A-V (2008). The stem cell marker CD133 (Prominin-1) is expressed in various human glandular epithelia. *Journal of Histochemistry and Cytochemistry*.

[15] Mizrak D, Brittan M, Alison MR (2008). CD133: molecule of the moment. *Journal of Pathology*.

[16] LeBoit PE, Weedon D, Sarasin A (2005). *Pathology and Genetics of Skin Tumours*.

[17] Chen S, Takahara M, Kido M (2008). Increased expression of an epidermal stem cell marker, cytokeratin 19, in cutaneous squamous cell carcinoma. *British Journal of Dermatology*.

[18] Bieniek R, Lazar AJF, Photopoulos C, Lyle S (2007). Sebaceous tumours contain a subpopulation of cells expressing the keratin 15 stem cell marker. *British Journal of Dermatology*.

[19] Fusi A, Ochsenreither S, Busse A, Rietz A, Keilholz U (2010). Expression of the stem cell marker nestin in peripheral blood of patients with melanoma. *British Journal of Dermatology*.

[20] Okudela K, Woo T, Mitsui H, Tajiri M, Masuda M, Ohashi K (2012). Expression of the potential cancer stem cell markers, CD133, CD44, ALDH1, and *β*-catenin, in primary lung adenocarcinoma—their prognostic significance. *Pathology International*.

[21] Zeppernick F, Ahmadi R, Campos B (2008). Stem cell marker CD133 affects clinical outcome in glioma patients. *Clinical Cancer Research*.

[22] Maw MA, Corbeil D, Koch J (2000). A frameshift mutation in prominin (mouse)-like 1 causes human retinal degeneration. *Human Molecular Genetics*.

[23] Pfenninger CV, Roschupkina T, Hertwig F (2007). CD133 is not present on neurogenic astrocytes in the adult subventricular zone, but on embryonic neural stem cells, ependymal cells, and glioblastoma cells. *Cancer Research*.

[24] Corbeil D, Röper K, Hellwig A (2000). The human AC133 hematopoietic stem cell antigen is also expressed in epithelial cells and targeted to plasma membrane protrusions. *Journal of Biological Chemistry*.

[25] Wang J, Sakariassen PØ, Tsinkalovsky O (2008). CD133 negative glioma cells form tumors in nude rats and give rise to CD133 positive cells. *International Journal of Cancer*.

[26] Ferrandina G, Martinelli E, Petrillo M (2009). CD133 antigen expression in ovarian cancer. *BMC Cancer*.

[27] Kemper K, Sprick MR, de Bree M (2010). The AC133 epitope, but not the CD133 protein, is lost upon cancer stem cell differentiation. *Cancer Research*.

